# Letter from Italy

**Published:** 1880-08

**Authors:** A. Lagorio

**Affiliations:** Chiavari, Italy


ARTICLE IX.

*Messrs. Editors:*—Rammatous hospital, founded in the year 1423, is to-day the largest and the oldest hospital of Genoa, Italy. Broad and high wards, good ventilation and plenty of light form its principal features. Although capable of holding nearly three thousand beds, another is in progress and almost completed, in a higher and healthier part of the city, at a cost of two millions of francs, kindly donated by the Duchess of Galliera, for such purpose. This is to be superior to any other hospital now existing in Italy, if not of Europe. The hospital abounds in plenty of material, medical as well as surgical and obstetrical, as is always found in the principal sea-ports. Clinics are given daily, and not being supplied with a suitable amphitheater the students are brought at the patient's bedside, where can be learned and studied fully and to better advantage the different ailments of mankind, many of which present special interest. I find in my notes a list of many interesting cases and opinions pronounced by the several professors, and selecting some of them I shall endeavor briefly to describe them to my medical colleagues.

The Medical Clinic is ably conducted by Prof. E. DeRenzi, the most talented and eloquent speaker of the faculty. During the past year he has presented at the clinic many remarkable cases, some of which he himself has published, and I have had occasion to note. Several have been cases of hemiplegia, and therefore a special attention was paid to the localization of the function of the faculty of language, for many patients presented a persistent or a temporary loss of speech. Such seat was determined by a series of uninterrupted studies and researches that began in our century with the phrenological doctrine of Gall, Spurzheim and Combe, to terminate lastly with the studies of Broca, of Tamburini and of Ferrier. About the beginning of this century Gall, Spurzheim and Combe admitted the seat of speech to the cerebral convolutions situated above the posterior portion of the supra-orbital lamina. In 1825 Bouillaud distinguished amnesic aphasia from ataxic, and laid the seat of the faculty of speech in the anterior lobes of the brain. In 1836 Dr. M. Dax demonstrated at the medical congress, held at Montpellier, that the faculty of speech does not occupy both the anterior lobes of the brain, but exclusively the left anterior. E. Broca in 1861 laid it in the posterior part of the third left frontal circonvolution. The studies of Dax's son, who admitted the lesion of aphasia to the anterior and external portion of the left middle lobe, does not modify radically the precedent doctrine. A case of permanent aphasia, and several of transitory aphasia were observed in clinics, and from them it is easy to make two deductions of no little interest for the localization of the faculty of speech.

A case of permanent aphasia was observed in a patient brought to the hospital deprived of speech and affected with right hemiplegia. The intelligence was disordered, he perceived tardily and imperfectly. By the movements and by the sounds that he uttered, he showed that he yet preserved the perceiving faculties. Sight intact, and the pupils reacted normally to the influence of light. The hemiplegia at the right was well marked, the muscles paralyzed and contracted. The muscles of the right side of the face were totally paralyzed and had lost their tonicity. Patient somewhat reacted under the remedies, but grew worse and lastly was found in a state of complete coma, flushed face and temperature much higher; this state lasted two days, until finally the patient died. At the autopsy were found two hæmorrhagic clots in the brain, seated in the left circumvolution of the corpus callosum, a centimeter and half behind its frontal extremity. The second corresponded to the external portion at the inferior extremity of the left ascendens parietal circumvolution, which was considerably larger than the right one. The same hæmorrhagic clot extended deeply in the median portion of the white matter of the cerebral hemisphere, and was situated in front and to the left, immediately beneath the island of Reil. This history which I have briefly described, shows in a clear and precise manner that a circumscribed lesion in the left cerebral hemisphere is sufficient, undoubtedly, to determine the loss of speech (aphasia). The seat of lesion in correspondence with the island of Reil, and at the point where pass the fibers arising from the posterior extremity of the third left frontal convolution, confirms the opinions of M. Dax and of Broca on the seat of the faculty of speech. In four other patients with cerebral hæmorrhage, in only one was found complete and permanent aphasia, and this is the above described case with right hemiplegia, and the apoplectic clot at the left. In other patients with temporary and incomplete aphasia, the hæmorrhagic lesion was found to the right, and the paralysis at the left. This sustains, to a certain degree, the opinion of Dax, who seated the seat of speech in the left cerebral hemisphere. A transitory aphasia hardly ever fails to manifest itself after the hæmorrhage in the right cerebral hemisphere that takes place near the psycomotor center of the facial. In private practice the professor said he had had occasion to notice complete aphasia, though transitory, in cases of hæmorrhage, which evidently had their seat in the right cerebral hemisphere. These cases, which are numerous and evident, bring us to the conclusion that the doctrine of Dax, Charcot, Falret, Broca, Hammond, etc., on the exclusive seat of the faculty of speech in the left cerebral hemisphere is incomplete. To assure ourselves in all clinical cases of permanent and transitory aphasia, complete and incomplete, we must admit two centers of the faculty of speech, situated one at the right and one at the left in symmetrical parts of the two cerebral hemispheres, the posterior part that is of the third frontal convolution, and the convolution of the island of Reil. The left center, in connection with the greater importance of the left cerebral hemisphere, requires a more considerable development and has a preponderating influence on the formation of speech, so that the destruction of the center of the seat of speaking, that is located in the right hemisphere, cannot cause the definitive and complete loss of such faculty; on the contrary, by the laws of compensation and adaption, which are most evident in physiological experiments and in the pathology of the cortical centers, the faculty of language very soon reappears normal as before. And respecting this the professor communicated an observation, not to be contradicted by further researches, because he had constantly verified it in numerous cases of cerebral hæmorrhage examined by him. That observation is that in all cases of hæmorrhage of the right cerebral hemisphere accompanied with marked paralysis of the facial, there exists also undoubtedly a temporary loss of speech. On the other hand, because of the greater development that the center of speech has in the left hemisphere, a lesion of such center produces the aphasia in a persistent and irreparable way. The predisposing influence of the left cerebral hemisphere in no other function shows itself more evident than in the formation of language. The explanation ordinarily given for this prevalence, not accounting the theories of Broca, Hammond, etc., consists in admitting that the prevalent use of the right hand is the cause of the greater development of the left hemisphere where terminate the nervous fibers of the right superior limb. Obernier says: (*Geschülste des Gehirns*) "Before learning how to call things with words, we habituate ourselves to indicate them with gestures of the right hand; moreover we learn with the indication of the right hand to spell words on tables, on books, and in consequence to designate things; and when in consecutive life we want to give brief commands, we express them not with words but with movements of the right hand, and when we want to speak with clearness we accompany the words with movements of the right hand; and lastly, in place of pronounced words we appropriate very often words written with the right hand."

Ferrier, in his *Pathological Illustrations of Brain Function*, speaks in the following terms: "The left hemisphere, as the right side of the body, is the conducting and impelling side; so that a lesion at the left side is the same as the loss of the right hand. A longer education is required to habituate the person to complete with the left hand all those delicate operations that could be completed easily with the right hand." Now that the education and the exercise of the right hand are not the only causes, nor the principle of the prevalence of the left hemisphere, the professor proved easily, by the history of a patient at the clinic, who was precisely left handed and therefore habituated to do all her delicate work with the left hand. In this case by the lesion of the right hemisphere, if the theory of Obernier, Ferrier, etc., were correct, we would have found a persistent aphasia, or at least greater than the one verified, because the prevalent action of the left hand, would have determined a predominant development in the right hemisphere. So that besides the prevalent exercise of the right hand we have another cause for the development of the faculty of speech in the left cerebral hemisphere, and this cause consists probably in a congenital or hereditary disposition. For many generations the exercise of the right hand provokes a larger development of the left hemisphere, and such development reproduces itself hereditarily in the same manner that in sons and nephews are reproduced some peculiarities that were noticed in the organic type of their forefathers. Only as favorable conditions to the development of the left hemisphere, but surely as principal causes of the seat of the faculty of speech in the same hemisphere, the professor also cited the greater quantity of blood and the precocious development of the left half of the brain.

Milk cure, grape cure and all other cures have been tried a good deal at this clinic, but at present I shall only describe the results obtained by the milk cure in cirrhosis of the liver, and acute articular rheumatism. In the former affection the results have been very encouraging, and is particularly commended while the disease is in its first stage. Its prognosis is considered by many authors, especially by Thierfelder, Lebert and Frierichs, fatal, death being inevitable. Jaccoud, Kunza, Bamberger, Surre, etc., have the same opinion, while F. Niemeyer says that diagnosticating the disease in its first stage we may attempt to arrest its progress. Most writers on disease of the liver rarely or never mention the milk cure, while a good deal of space is wasted in speaking of other remedies, which daily experience has shewn to be inefficient or even hurtful. Milk in cases of cirrhosis is one of the best of remedies, and when early used has given in this clinic many curative results. It increases the urinary secretion, almost to a true polyuria, takes away the venous stasis and provokes the disappearance of the ascites and of the œdema of the inferior extremities. Moreover it is an aliment easily digested and overcomes the globular anæmia. In this manner keeping up the general condition of the patient, the collateral circulation is developed.

Acute articular rheumatism has been subjected to a like cure. Tripier, Besnier and Biot have recognized the value of the milk diet to overcome the affection. Since 1869 Dr. Tripier, of Lyons, has used the lacteal cure as the most efficacious remedy in rheumatism, and ever since has used it with marked success. This year Dr. Biot has published in the *Revue Mensuelle de Médecine et de Chirurgie*, a long article in which he demonstrates with many practical arguments, that milk rapidly shortens the term of treatment, and in the space of three to eight days calms the rheumatic pains. Moreover, milk increases the quantity of urine and the secretions of all salines, the urates particularly. This treatment must be used only in acute articular rheumatism; the sooner the better. Salicylate of soda is used with it, and the professor thinks it opportune to use the two remedies contemporaneously. The milk with the salicylic acid will correct the effects of the disease and of the remedy, which latter are favorable to the development of globular anæmia. Tripier finds no contra-indication in the contemporaneous use of the remedies. He, however, while attributing the principal action to the milk, recognizes the salicylic acid only as an analgesic. In fact the acid, besides its sedative action on the pain, has an important influence on the fever, which is one of the principal elements of the disease; therefore it will be useful to prescribe at the same time the salycilic acid and the lacteal treatment. Wishing to use only one of the remedies, Prof. DeRenzi thinks the lacteal treatment is to be preferred by all means to the salicylic acid, when the articular rheumatism is associated with some heart affection.

A. LAGORIO, M.D.

CHIAVARI, Italy, June 1, 1880.

